# *N*-[(*E*)-4-Bromo­benzyl­idene]-3,4-di­methyl­aniline

**DOI:** 10.1107/S1600536813008143

**Published:** 2013-04-05

**Authors:** Li-Xia Sun, Ling-Zhi Zhu, Jun-Kai Wang

**Affiliations:** a College of Materials Science & Engineering, China Jiliang University, Hangzhou 310018, People’s Republic of China

## Abstract

In the title compound, C_15_H_14_BrN, the dihedral angle between the benzene rings is 6.4 (2)° and the mol­ecule has an *E* conformation about the C=N bond. In the crystal, mol­ecules are linked by C—H⋯π inter­actions, forming two-dimensional networks lying parallel to (001).

## Related literature

Schiff bases derivatives have many pharmaceutical activities. For their anti­fungal properties, see: Aziz *et al.* (2010 ▶), for their radical scavenging activity, see: Lu *et al.* (2012 ▶), for their inhibition of enzyme activity, see: Schmidt *et al.* (2009 ▶) and for their anti­bacterial activity, see: Shi *et al.* (2010 ▶). For related structures, see: Sun *et al.* (2011*a* ▶,*b* ▶); Guo *et al.* (2011 ▶). For standard bond lengths, see: Allen *et al.* (1987 ▶).
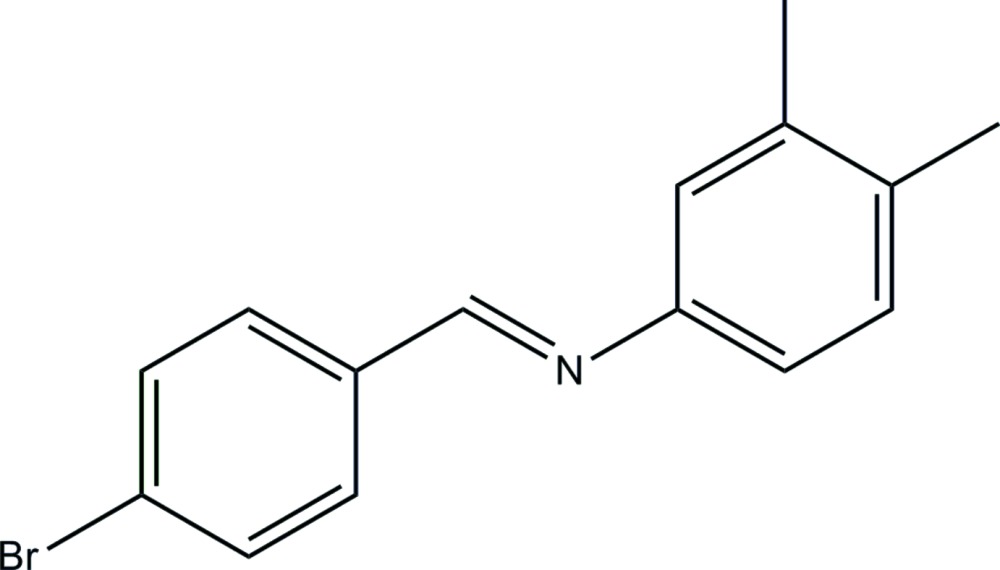


## Experimental

### 

#### Crystal data

C_15_H_14_BrN
*M* *_r_* = 288.18
Orthorhombic, 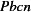

*a* = 14.868 (7) Å
*b* = 6.161 (3) Å
*c* = 28.609 (13) Å
*V* = 2621 (2) Å^3^
*Z* = 8
Mo *K*α radiation
μ = 3.11 mm^−1^
*T* = 296 K
0.22 × 0.19 × 0.14 mm

#### Data collection

Bruker APEXII CCD diffractometer
Absorption correction: multi-scan (*SADABS*; Sheldrick, 1996 ▶) *T*_min_ = 0.547, *T*_max_ = 0.670
17312 measured reflections
2445 independent reflections
1438 reflections with *I* > 2σ(*I*)
*R*_int_ = 0.090

#### Refinement

*R*[*F* ^2^ > 2σ(*F* ^2^)] = 0.058
*wR*(*F* ^2^) = 0.148
*S* = 1.03
2445 reflections
156 parameters
H-atom parameters constrained
Δρ_max_ = 0.55 e Å^−3^
Δρ_min_ = −0.56 e Å^−3^

### 

Data collection: *APEX2* (Bruker, 2004 ▶); cell refinement: *SAINT* (Bruker, 2004 ▶); data reduction: *SAINT*; program(s) used to solve structure: *SHELXS97* (Sheldrick, 2008 ▶); program(s) used to refine structure: *SHELXL97* (Sheldrick, 2008 ▶); molecular graphics: *SHELXTL* (Sheldrick, 2008 ▶); software used to prepare material for publication: *SHELXTL*.

## Supplementary Material

Crystal structure: contains datablock(s) global, I. DOI: 10.1107/S1600536813008143/su2579sup1.cif

Structure factors: contains datablock(s) I. DOI: 10.1107/S1600536813008143/su2579Isup2.hkl

Supplementary material file. DOI: 10.1107/S1600536813008143/su2579Isup3.cml

Additional supplementary materials:  crystallographic information; 3D view; checkCIF report

## Figures and Tables

**Table 1:** Hydrogen-bond geometry (Å, °) *Cg*1 and *Cg*2 are the centroids of the C1–C6 and C8–C13 rings, respectively.

*D*—H⋯*A*	*D*—H	H⋯*A*	*D*⋯*A*	*D*—H⋯*A*
C9—H9⋯*Cg*1^i^	0.93	2.99	3.773 (5)	143
C12—H12⋯*Cg*2^ii^	0.93	2.77	3.507 (5)	137

Symmetry codes: (i) 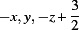; (ii) 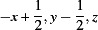.

**Fig. 1.**
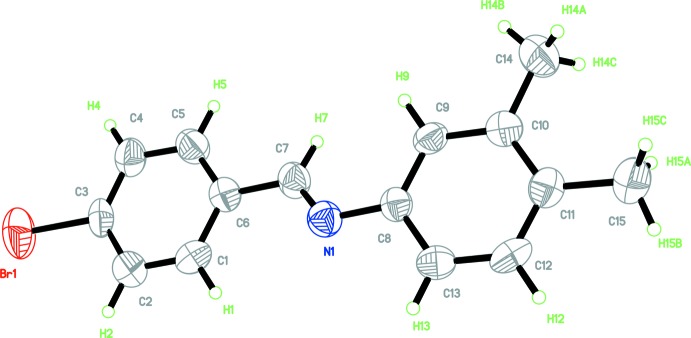
The molecular structure of the title molecule, with atom labelling. The displacement ellipsoids are drawn at the 50% probability level.

## supplementary crystallographic information

### Comment

Schiff base ligands have received much attention during past years. They have many pharmaceutical activities, such as antifungal effects (Aziz *et al.*, 2010), radical scavenging activity (Lu *et al.*, 2012), inhibition of enzyme activity (Schmidt *et al.*, 2009), antibacterial activities (Shi *et al.*, 2010). We report herein on the crystal structure of the new title Schiff base compound.

In the title molecule, Fig. 1, the bond lengths (Allen *et al.*, 1987) and angles are normal and comparable to the values observed in similar compounds (Sun *et al.*, 2011*a*,*b*; Guo *et al.*, 2011). The dihedral angle between the two aromatic rings in the Schiff base molecule is 6.4 (2) °, indicating that two these rings are approximatively coplanar. The molecule has an E conformation about the C7═N1 bond.

In the crystal, molecules are linked by C-H···π interactions (Table 1).

### Experimental

A mixture of 4-bromobenzaldehyde (5 mmol), 3,4-dimethylaniline (5 mmol) and methanol (50 ml) was refluxed for 6 h. It was then allowed to cool and was filtered. Recrystallization of the crude product from methanol yielded yellow block-like crystals.

### Refinement

H atoms were positioned geometrically and refined using the riding-model approximation: C—H = 0.93–0.96 Å with *U*_iso_(H) = 1.5*U*_eq_(C-methyl) and = 1.2*U*_eq_(C) for other H atoms.

### Crystal data

**Table:**

C_15_H_14_BrN	*F*(000) = 1168
*M**_r_* = 288.18	*D*_x_ = 1.461 Mg m^−^^3^
Orthorhombic, *P**b**c**n*	Mo *K*α radiation, λ = 0.71073 Å
Hall symbol: -P 2n 2ab	Cell parameters from 2443 reflections
*a* = 14.868 (7) Å	θ = 2.7–25.5°
*b* = 6.161 (3) Å	µ = 3.11 mm^−^^1^
*c* = 28.609 (13) Å	*T* = 296 K
*V* = 2621 (2) Å^3^	Block, yellow
*Z* = 8	0.22 × 0.19 × 0.14 mm

### Data collection

**Table:**

Bruker APEXII CCD diffractometer	2445 independent reflections
Radiation source: fine-focus sealed tube	1438 reflections with *I* > 2σ(*I*)
Graphite monochromator	*R*_int_ = 0.090
φ and ω scans	θ_max_ = 25.5°, θ_min_ = 2.7°
Absorption correction: multi-scan (*SADABS*; Sheldrick, 1996)	*h* = −18→18
*T*_min_ = 0.547, *T*_max_ = 0.670	*k* = −7→7
17312 measured reflections	*l* = −32→34

### Refinement

**Table:**

Refinement on *F*^2^	Primary atom site location: structure-invariant direct methods
Least-squares matrix: full	Secondary atom site location: difference Fourier map
*R*[*F*^2^ > 2σ(*F*^2^)] = 0.058	Hydrogen site location: inferred from neighbouring sites
*wR*(*F*^2^) = 0.148	H-atom parameters constrained
*S* = 1.03	*w* = 1/[σ^2^(*F*_o_^2^) + (0.0449*P*)^2^ + 6.739*P*] where *P* = (*F*_o_^2^ + 2*F*_c_^2^)/3
2445 reflections	(Δ/σ)_max_ = 0.001
156 parameters	Δρ_max_ = 0.55 e Å^−^^3^
0 restraints	Δρ_min_ = −0.56 e Å^−^^3^

### Special details

**Table:**

Geometry. Bond distances, angles etc. have been calculated using the rounded fractional coordinates. All su's are estimated from the variances of the (full) variance-covariance matrix. The cell esds are taken into account in the estimation of distances, angles and torsion angles
Refinement. Refinement of *F*^2^ against ALL reflections. The weighted *R*-factor *wR* and goodness of fit *S* are based on *F*^2^, conventional *R*-factors *R* are based on *F*, with *F* set to zero for negative *F*^2^. The threshold expression of *F*^2^ > σ(*F*^2^) is used only for calculating *R*-factors(gt) *etc*. and is not relevant to the choice of reflections for refinement. *R*-factors based on *F*^2^ are statistically about twice as large as those based on *F*, and *R*- factors based on ALL data will be even larger.

### Fractional atomic coordinates and isotropic or equivalent isotropic displacement parameters (Å^2^)

**Table:**

	*x*	*y*	*z*	*U*_iso_*/*U*_eq_	
Br1	0.12941 (5)	1.15486 (14)	0.55818 (2)	0.0821 (3)	
N1	0.1277 (3)	0.5957 (7)	0.76719 (15)	0.0460 (16)	
C1	0.1549 (3)	0.7560 (9)	0.6734 (2)	0.0507 (19)	
C2	0.1579 (4)	0.8357 (11)	0.6281 (2)	0.058 (2)	
C3	0.1256 (3)	1.0405 (10)	0.62021 (17)	0.0460 (19)	
C4	0.0911 (4)	1.1681 (10)	0.65516 (19)	0.0533 (19)	
C5	0.0907 (4)	1.0843 (9)	0.70019 (19)	0.0523 (19)	
C6	0.1210 (3)	0.8791 (9)	0.70960 (18)	0.0417 (17)	
C7	0.1164 (3)	0.7919 (9)	0.75771 (19)	0.0470 (19)	
C8	0.1253 (3)	0.5192 (8)	0.81399 (16)	0.0387 (16)	
C9	0.0878 (3)	0.6300 (8)	0.85173 (18)	0.0430 (17)	
C10	0.0929 (3)	0.5490 (9)	0.89694 (18)	0.0440 (17)	
C11	0.1357 (3)	0.3504 (9)	0.90483 (19)	0.0477 (17)	
C12	0.1714 (3)	0.2393 (9)	0.8668 (2)	0.0477 (19)	
C13	0.1656 (3)	0.3213 (8)	0.8222 (2)	0.0443 (17)	
C14	0.0523 (5)	0.6765 (11)	0.93710 (19)	0.072 (3)	
C15	0.1459 (4)	0.2609 (11)	0.9540 (2)	0.070 (2)	
H1	0.17610	0.61680	0.67950	0.0610*	
H2	0.18130	0.75260	0.60390	0.0690*	
H4	0.06880	1.30610	0.64890	0.0640*	
H5	0.06940	1.16980	0.72460	0.0630*	
H7	0.10450	0.88760	0.78210	0.0560*	
H9	0.05860	0.76120	0.84650	0.0520*	
H12	0.19980	0.10660	0.87160	0.0570*	
H13	0.18910	0.24250	0.79730	0.0530*	
H14A	0.00220	0.59760	0.94980	0.1080*	
H14B	0.03200	0.81510	0.92590	0.1080*	
H14C	0.09680	0.69750	0.96100	0.1080*	
H15A	0.17400	0.36800	0.97350	0.1050*	
H15B	0.18250	0.13260	0.95320	0.1050*	
H15C	0.08770	0.22570	0.96640	0.1050*	

### Atomic displacement parameters (Å^2^)

**Table:**

	*U*^11^	*U*^22^	*U*^33^	*U*^12^	*U*^13^	*U*^23^
Br1	0.0956 (5)	0.1062 (6)	0.0446 (4)	−0.0111 (5)	−0.0052 (3)	0.0232 (4)
N1	0.045 (2)	0.049 (3)	0.044 (3)	−0.001 (2)	−0.001 (2)	0.000 (2)
C1	0.049 (3)	0.044 (3)	0.059 (4)	0.009 (3)	−0.003 (3)	0.004 (3)
C2	0.059 (3)	0.067 (5)	0.047 (4)	0.007 (3)	0.000 (3)	−0.004 (3)
C3	0.044 (3)	0.063 (4)	0.031 (3)	−0.008 (3)	−0.007 (2)	0.009 (3)
C4	0.065 (3)	0.049 (4)	0.046 (3)	−0.002 (3)	−0.008 (3)	0.006 (3)
C5	0.060 (3)	0.052 (4)	0.045 (3)	0.001 (3)	0.001 (3)	−0.004 (3)
C6	0.041 (3)	0.043 (3)	0.041 (3)	−0.007 (3)	−0.001 (2)	−0.001 (2)
C7	0.052 (3)	0.047 (4)	0.042 (3)	−0.003 (3)	−0.007 (2)	−0.005 (3)
C8	0.037 (2)	0.041 (3)	0.038 (3)	−0.003 (3)	−0.002 (2)	0.000 (2)
C9	0.043 (3)	0.037 (3)	0.049 (3)	0.008 (2)	0.001 (2)	0.004 (3)
C10	0.041 (3)	0.042 (3)	0.049 (3)	−0.004 (3)	−0.003 (2)	−0.005 (3)
C11	0.046 (3)	0.047 (3)	0.050 (3)	−0.004 (3)	−0.004 (3)	0.006 (3)
C12	0.036 (3)	0.035 (3)	0.072 (4)	0.005 (2)	−0.002 (3)	0.006 (3)
C13	0.042 (3)	0.039 (3)	0.052 (3)	0.000 (2)	0.003 (2)	−0.003 (3)
C14	0.090 (5)	0.076 (5)	0.050 (4)	0.013 (4)	0.004 (3)	−0.008 (3)
C15	0.075 (4)	0.073 (4)	0.061 (4)	0.012 (3)	−0.002 (3)	0.017 (3)

### Geometric parameters (Å, º)

**Table:**

Br1—C3	1.910 (5)	C11—C15	1.519 (8)
N1—C7	1.250 (7)	C12—C13	1.375 (8)
N1—C8	1.420 (6)	C1—H1	0.9300
C1—C2	1.387 (8)	C2—H2	0.9300
C1—C6	1.379 (8)	C4—H4	0.9300
C2—C3	1.369 (9)	C5—H5	0.9300
C3—C4	1.372 (8)	C7—H7	0.9300
C4—C5	1.388 (8)	C9—H9	0.9300
C5—C6	1.369 (8)	C12—H12	0.9300
C6—C7	1.479 (8)	C13—H13	0.9300
C8—C9	1.394 (7)	C14—H14A	0.9600
C8—C13	1.379 (7)	C14—H14B	0.9600
C9—C10	1.388 (7)	C14—H14C	0.9600
C10—C11	1.398 (8)	C15—H15A	0.9600
C10—C14	1.517 (8)	C15—H15B	0.9600
C11—C12	1.391 (8)	C15—H15C	0.9600
			
C7—N1—C8	121.5 (5)	C1—C2—H2	121.00
C2—C1—C6	121.3 (5)	C3—C2—H2	121.00
C1—C2—C3	118.0 (5)	C3—C4—H4	121.00
Br1—C3—C2	118.9 (4)	C5—C4—H4	121.00
Br1—C3—C4	118.5 (4)	C4—C5—H5	119.00
C2—C3—C4	122.6 (5)	C6—C5—H5	119.00
C3—C4—C5	117.7 (5)	N1—C7—H7	118.00
C4—C5—C6	121.7 (5)	C6—C7—H7	118.00
C1—C6—C5	118.7 (5)	C8—C9—H9	119.00
C1—C6—C7	121.1 (5)	C10—C9—H9	119.00
C5—C6—C7	120.2 (5)	C11—C12—H12	119.00
N1—C7—C6	123.2 (5)	C13—C12—H12	119.00
N1—C8—C9	125.3 (4)	C8—C13—H13	120.00
N1—C8—C13	116.3 (4)	C12—C13—H13	120.00
C9—C8—C13	118.4 (5)	C10—C14—H14A	109.00
C8—C9—C10	121.6 (5)	C10—C14—H14B	109.00
C9—C10—C11	119.3 (5)	C10—C14—H14C	110.00
C9—C10—C14	119.9 (5)	H14A—C14—H14B	109.00
C11—C10—C14	120.8 (5)	H14A—C14—H14C	109.00
C10—C11—C12	118.6 (5)	H14B—C14—H14C	110.00
C10—C11—C15	120.9 (5)	C11—C15—H15A	109.00
C12—C11—C15	120.5 (5)	C11—C15—H15B	109.00
C11—C12—C13	121.4 (5)	C11—C15—H15C	110.00
C8—C13—C12	120.7 (5)	H15A—C15—H15B	109.00
C2—C1—H1	119.00	H15A—C15—H15C	109.00
C6—C1—H1	119.00	H15B—C15—H15C	109.00
			
C7—N1—C8—C13	−160.1 (5)	C5—C6—C7—N1	166.9 (5)
C8—N1—C7—C6	178.2 (4)	N1—C8—C9—C10	−176.2 (4)
C7—N1—C8—C9	18.2 (7)	C13—C8—C9—C10	2.1 (7)
C2—C1—C6—C5	−0.6 (8)	N1—C8—C13—C12	176.2 (4)
C6—C1—C2—C3	−0.4 (8)	C9—C8—C13—C12	−2.3 (7)
C2—C1—C6—C7	178.9 (5)	C8—C9—C10—C11	−0.8 (7)
C1—C2—C3—Br1	179.7 (4)	C8—C9—C10—C14	179.1 (5)
C1—C2—C3—C4	0.2 (8)	C9—C10—C11—C12	−0.4 (7)
Br1—C3—C4—C5	−178.6 (4)	C9—C10—C11—C15	177.5 (5)
C2—C3—C4—C5	0.9 (8)	C14—C10—C11—C12	179.7 (5)
C3—C4—C5—C6	−1.9 (9)	C14—C10—C11—C15	−2.4 (7)
C4—C5—C6—C1	1.8 (8)	C10—C11—C12—C13	0.3 (7)
C4—C5—C6—C7	−177.7 (5)	C15—C11—C12—C13	−177.7 (5)
C1—C6—C7—N1	−12.5 (7)	C11—C12—C13—C8	1.1 (7)

### Hydrogen-bond geometry (Å, º)

Cg1 and Cg2 are the centroids of the C1–C6 and C8–C13 rings, respectively.

**Table:**

*D*—H···*A*	*D*—H	H···*A*	*D*···*A*	*D*—H···*A*
C9—H9···*Cg*1^i^	0.93	2.99	3.773 (5)	143
C12—H12···*Cg*2^ii^	0.93	2.77	3.507 (5)	137

Symmetry codes: (i) −*x*, *y*, −*z*+3/2; (ii) −*x*+1/2, *y*−1/2, *z*.
